# Germline genome editing of human IVF embryos should not be subject to overly stringent restrictions

**DOI:** 10.1007/s10815-024-03174-x

**Published:** 2024-07-05

**Authors:** Kevin Richard Smith

**Affiliations:** Division of Health Science, School of Applied Sciences, Abertay University, Dundee, DD1 1HG UK

**Keywords:** CRISPR-Cas9, Gene editing, Gene therapy, Germline genome editing, Medical ethics, Pre-implantation genetic testing

## Abstract

This paper critiques the restrictive criteria for germline genome editing recently proposed by Chin, Nguma, and Ahmad in this journal. While praising the authors for resisting fervent calls for an outright ban on clinical applications of the technology, this paper argues that their approach is nevertheless unduly restrictive, and may thus hinder technological progress. This response advocates for weighing potential benefits against risks without succumbing to excessive caution, proposing that ethical oversight combined with genetic scrutiny at the embryo stage post-editing can enable responsible use of the technology, ultimately reducing the burden of genetic diseases and enhancing human health, akin to how IVF transformed reproductive medicine despite strong initial opposition.

## Introduction

In this issue of this JARG, Chin, Nguma, and Ahmad proposed a set of criteria that they asserted ought to be applied to future clinical trials of germline genome editing [1]. This is a most germane time to consider this controversial topic because it is 5 years since Chinese scientist He Jiankui announced the world’s first ever attempt to create germline genetically modified humans. This experiment was met with intense international disapprobation, leading to He’s downfall and imprisonment, and resulting in a 5-year international moratorium on human germline genome editing [2].

Chin, Nguma, and Ahmad (henceforth referred to as “the authors” for the sake of brevity) wrote their criteria on the basis that this moratorium is about to expire. In so doing, they expressed a somewhat overly cautious perspective on the topic. Writing as a medical ethicist, in the present paper, I will argue that the authors’ criteria for permitting future human germline genome editing attempts are unduly restrictive, and if adopted will tend to stifle a nascent science that holds great potential benefits for humanity. I shall start by considering the various arguments made by the authors, then address the specific criteria that they have proposed, and finally conclude on a more permissive note than that of the authors.

## Is germline genome editing “really necessary”?

The authors point out that human germline genome editing of IVF embryos differs from somatic editing aimed at treating current patients with severe diseases because it is intended to prevent genetic diseases in future generations. They argue that due to insufficient safety data, it is premature to edit embryos’ genes, especially when alternatives like adoption, donor gametes, or remaining child-free exist. They do accept, however, that cultural attitudes, particularly in some non-Western countries, make germline editing a more appealing option for those seeking genetically related children.

In response to this, it should first be noted that the desire for genetically related children is a strong and nearly universal sentiment among prospective parents [3, 4]; it is not merely a niche preference, as the authors seem to imply. In most models of medical ethics, as well as in everyday morality, it is generally held to be a moral good—if not an imperative—to permit and facilitate competent adults to satisfy their deeply held preferences, as long as by so doing others are not harmed in the process. Therefore, insofar as there may be a latent demand for germline genome editing of IVF embryos as a means to allow prospective parents to have healthy genetically related offspring, it would seem prima facie morally correct to support the development of this technology.

It should also be noted that in many jurisdictions, genetic affinity and blood ties have been used as the basis for family court case verdicts. Legal precedents in countries such as Singapore, the USA, and the UK emphasize that an individual’s desire to have a child carrying their genes is a basic human impulse. Courts recognize that in the “ordinary human experience,” parents and children are typically related by blood and share physical traits, reflecting deep societal norms and personal identities [5, 6, 7, 8].

## Counseling of couples on the safety risks of germline genome editing

The authors point out that couples contemplating germline genome editing of IVF embryos must be appraised of the risks involved. This is uncontroversial: informed consent is a cornerstone of medical ethics. The authors then follow up with a long list of “safety flaws” of genome editing with CRISPR-Cas9 technology, which may be summarized as mutational damage and mosaicism.

In response, it should be said firstly that while in the initial stages of genome editing adverse effects occurred quite frequently, the precision of CRISPR has been significantly enhanced through rigorous experimental efforts, with numerous recent studies demonstrating minimal CRISPR-Cas9-related damage in cells, embryos, and animals (see for example [9, 10]). Furthermore, any ethically approved genome editing endeavor would include necessary genetic tests, entailing an embryo created through genome editing undergoing thorough pre-implantation testing (PGT) to detect and discard any embryos with issues. With current cutting-edge PGT technology, up to 5–10 cells can be safely biopsied from the trophectoderm of a blastocyst-stage embryo, and the genome of each cell can be analyzed using single-cell whole genome sequencing (scWGS).

More fundamentally, the important question must be this: What is the *magnitude* of the risk? Unfortunately, the authors fail to provide any quantification in this regard. While there are some genuine risks from genome editing, it cannot be sufficient to claim that simply because adverse events sometimes occur, we must therefore stringently restrict germline genome editing. Instead, a rational analysis requires these risks be set against two countervailing factors: [a] the background mutational damage in the genome, and [b] the potential health benefits from the technology. In terms of [a], current estimates suggest that the overall germline mutation rate is about 70 new mutations per generation, meaning that, on average, each person is likely to carry around 70 genetic changes that were not found in the genomes of either parent [11, 12, 13]. Given the sparsity of crucial genetic sequences in the genome, and the phenomena of haplosufficiency and bypass suppression, most of these mutations will have no detectable deleterious effects. Nevertheless, around 1% of newborns are born with a significant monogenetic defect, and an additional 2% of newborns exhibit a congenital malformation—a considerable number of which are due to de novo mutations [14, 15, 16, 17].

Of course, any additional mutational risk from CRISPR-Cas9 technology is unwelcome, even in the context of a much higher natural background mutational burden. And this added risk would give some reason to proscribe germline genetic modification, if it were not for [b] above—the potential health benefits of germline editing. If the genome of one (or both) prospective parents contains a gene that causes a specific disorder, a tailored editing strategy could permanently modify this gene in an embryo from these parents, thereby removing the problematic sequence from the child’s genome, and preventing the manifestation of the genetic disorder. Of particular importance is the fact that, once eliminated, the problematic gene would no longer threaten descendants of the individual so modified; additionally, its frequency would reduce within the population. These are major positives: any rational assessment of germline genome editing requires a risk vs benefit analysis that considers not only the negatives (i.e., mutations and mosaicism) but also the positives [18]. The authors have not presented such an analysis.

## PGT as an alternative to germline genome editing?

The authors suggest that because pre-implantation genetic testing (PGT) is a mature and provenly safe technology, it obviates the need for germline genomic editing in most cases. It is indeed true that PGT is currently the only method available to detect genetic defects in embryos, making it a crucial tool for preventing genetic disorders [19]. And it should be used as a front-line approach wherever it will be effective. However, its application is restricted. Firstly, it can only detect well-defined monogenic disorders. Secondly, it is limited by the number of available embryos from prospective parents; consequently, PGT is not suitable for polygenic conditions or multiple genetic disorders, as finding a single “disease-free” embryo would require an impractically large number of embryos. Additionally, PGT is not useful in cases where both parents are homozygous for a recessive disease-causing allele or one parent is homozygous for a dominant disease-causing allele.

In contrast, genome editing, particularly through tools like CRISPR, offers the potential to not just identify but correct harmful genetic sequences directly within embryos. Initially, this approach could be applied to prevent specific monogenic disorders, particularly in situations where PGT is ineffective. As genome editing technology advances, its application could expand to tackle other genetic issues. Although addressing polygenic and multiple disorders through genome editing is a future (as opposed to present-day) possibility, progress in this direction depends on initiating the use of genome editing for monogenic conditions [18]. Only by doing so will we enable the technology to develop rapidly—which is an ethical imperative, given its great scope for benefitting future people. In focusing narrowly on PGT technology, the authors fail to factor-in the crucial importance of promoting technological development of germline genome editing by in some cases utilizing the technology even where PGT would be applicable.

## Prohibition of “non-essential” genome editing?

The authors argue that the ancillary use of germline genome editing in non-critical cases, where alternatives exist, may be difficult to justify due to substantial medical risks. Specifically, they deem it unwarranted to edit heterozygous IVF embryos for a recessive gene mutation because such embryos could still produce unaffected offspring. Moreover, they suggest that editing affected embryos simply to increase the pool of viable embryos for transfer is not justified, given the risks and the possibility of undertaking additional IVF cycles, and potential higher costs depending on subsidies or insurance.

However, this stance overlooks several critical factors. Firstly, while a heterozygous individual may appear phenotypically unaffected, they still carry a deleterious gene. Germline genome editing offers a permanent solution by eliminating this gene, which not only ensures the health of the immediate offspring but also secures a healthier genetic future for subsequent generations. This broader perspective on public health genetics argues strongly for the proactive use of genome editing.

Furthermore, concerns regarding the high costs associated with germline genome editing are likely to diminish over time as the technology advances and becomes more widespread. Consider the development of computers: once prohibitively expensive but now widely affordable. This general trend towards lower costs as technology develops suggests a similar trajectory for genome editing technologies, as the initial high investment costs are likely to be offset by the technology’s increasing accessibility and efficiency.

Therefore, the argument against the use of genome editing based on current medical risks and costs fails to account for the potential long-term benefits and advancements in technology. Allowing the technology to be used and developed should pave the way for safer, more affordable, and more effective treatments, outweighing initial hesitations about its application and cost. In light of these considerations, the stance taken by the authors ought to be seen as overly cautious, potentially hindering progress in genetic medicine that could benefit future generations significantly.

## Criteria for use of germline genome editing

The authors proposed a set of six “stringent criteria” that they claim ought to be fulfilled before any future human experiments with germline genomic editing may be countenanced. In summary form, these are:

1. Germline genome editing should be *cautiously and judiciously applied* while avoiding *non-essential usage*, and couples should be *rigorously* *counselled* on risks and alternatives.
2. Genome editing should only be performed on the entire batch of the patient’s IVF embryos without initial PGT screening if all of them are expected to be affected by genetic disease.
3. Where some embryos might be unaffected, the batch should be screened with PGT to determine which of these can potentially give rise to an unaffected child, which will then be selected for transfer to the patient.
4. IVF embryos screened by PGT to be heterozygous for recessive gene mutations (carrier status) should not be subjected to germline genome editing.
5. If patients fail to conceive with unaffected embryos or none are identified after PGT screening, they should undergo a new IVF cycle rather than editing remaining affected embryos.
6. Genome editing of remaining affected embryos may be permitted as a last resort only if a new IVF cycle fails to produce unaffected embryos due to advanced maternal age or diminished ovarian reserves.

Criterion 1 is uncontentious: cautious and judicious application of any medical treatment or technology is always wise and inherently an ethical imperative. Furthermore, it is essential that patients receive thorough counseling to ensure informed consent, which is an inviolable ethical requirement.

The remaining Criteria 2–6 do not aim to completely prohibit germline genome editing, which is very encouraging for those advocating for the advancement of this technology—a development that could potentially greatly benefit future patients and generations. Nevertheless, many will nonetheless find these criteria overly restrictive. The authors justify their approach by citing the current uncertainties and safety concerns surrounding germline genome editing. Although this caution may seem reasonable at first glance, it represents a conservative stance that could hinder progress in this promising field. Ethicists, including myself, have critiqued such precautionary measures as fundamentally flawed and, therefore, unsuitable for germline genome editing [18, 20].

The authors go on to point out that germline genome editing straddles a delicate line between preventing genetic diseases and the potential for human enhancement, such as increased immunity or elevated IQ. In so doing, they convey a common view—the notion that while therapeutic uses of the technology may be acceptable or even desirable, “enhancement” uses are definitely unacceptable [21]. A debate on this (futuristic) topic is beyond the scope of the present paper; however, it is worth noting that several ethicists view at least some forms of genetic enhancement as being of potential value to humanity and thus justifiable on ethical grounds. For example, it would arguably be a good thing if all people (or as many people as possible) were modified to have excellent eyesight or better memory capacities [22].

## Concluding remarks

He Jiankui’s provocative work 5 years ago in germline genomic editing was not the first time a medical scientist has pushed ahead with controversial but potentially revolutionary technology in reproductive medicine. Robert Edwards’s pioneering work in developing in vitro fertilization (IVF) technology provides a valuable analogy. In 1978, the World celebrated the birth of Louise Joy Brown, the first successful IVF baby. Notwithstanding strong and persistence criticism from conservative commentators, this breakthrough was heralded as one of the most remarkable medical accomplishments of the twentieth century. Robert Edwards was later awarded the Nobel Prize in Physiology or Medicine in 2010 for this revolutionary contribution, which has since transformed infertility treatment and conferred immense benefits to humanity [23, 24].

However, it is crucial to consider the potential outcome had Edwards’s initial experiments failed, especially in today’s climate of heightened caution towards medical technology. Much like Edwards, He Jiankui aimed to be a pioneer, albeit in a more ethically complex and globally scrutinized environment. But the latter’s experiment never had the chance to produce the kind of clearcut triumph that IVF did: the birth of a baby from an infertile woman was an obvious and instantly recognizable triumph, whereas the infants born following He Jiankui’s work could only benefit in the future (from possible HIV resistance)—so this was not an instantly recognizable accomplishment.

In the event, He Jiankui’s experiment was met with near-unanimous severe condemnation by scientists and other commentators. This negative attention led to intense scrutiny of his work, which revealed breaches of human subjects research regulations and led to his subsequent imprisonment. Yet the infants born through his work were apparently healthy. By producing the world’s first demonstration of germline gene editing in humans, He Jiankui made a revolutionary advancement that was as groundbreaking as, if not more so than, the development of human IVF.

The reaction to He Jiankui’s work suggests a prevailing knee-jerk response against germline genome editing, driven by a precautionary approach that may stifle innovation in this promising field. While caution is necessary, it must be balanced with rational risk–benefit analysis to avoid impeding technological progress that could potentially revolutionize healthcare and provide unprecedented benefits to future generations.

In drafting their Criteria, I suggest that the authors Chin, Nguma, and Ahmad have done well to avoid following the rather hysterical demands for a permanent ban on germline genome editing that some scientists and ethicists and others called for following the revelation of He Jiankui’s experiments. Nevertheless, their position is still arguably overly cautious. Genome editing holds the potential to transform reproductive and genetic medicine. While it carries some risks, coupled with pre-screening of edited embryos, CRISPR-Cas technology has developed to the point where it can support well-designed protocols that target specific monogenic disorders. There are no sound reasons to delay the use of genome editing due to excessive caution. Starting genome editing sooner will allow future children to experience its benefits earlier. As the practice becomes more widespread, we can expect rapid technological advancements, leading to future generations of people benefitting from avoidance of, or reduced susceptibility to, the myriad of disorders that presently afflict humanity.
